# Chemical Composition, Fatty Acid and Mineral Content of Food-Grade White, Red and Black Sorghum Varieties Grown in the Mediterranean Environment

**DOI:** 10.3390/foods11030436

**Published:** 2022-02-02

**Authors:** Paola Pontieri, Jacopo Troisi, Matteo Calcagnile, Scott R. Bean, Michael Tilley, Fadi Aramouni, Antonio Boffa, Giacomo Pepe, Pietro Campiglia, Fabio Del Giudice, Alberto L. Chessa, Dmitriy Smolensky, Mariarosaria Aletta, Pietro Alifano, Luigi Del Giudice

**Affiliations:** 1 Istituto di Bioscienze e BioRisorse-UOS Napoli-CNR c/o Dipartimento di Biologia, Sezione di Igiene, 80134 Napoli, Italy; boffa83@gmail.com (A.B.); luigi.delgiudice@ibbr.cnr.it (L.D.G.); 2 Theoreosrl, Spin Off of the University of Salerno, Department of Medicine and Surgery, Via Degli Ulivi, 3, Montecorvino Pugliano, 84090 Salerno, Italy; troisi@theoreosrl.com; 3 Dipartimento di Scienze e Tecnologie Biologiche e Ambientali, Università del Salento, 73100 Lecce, Italy; matteo.calcagnile@unisalento.it (M.C.); pietro.alifano@unisalento.it (P.A.); 4 USDA-ARS, CGAHR, Manhattan, KS 66502, USA; scott.bean@usda.gov (S.R.B.); michael.tilley@usda.gov (M.T.); fadi.aramouni@usda.gov (F.A.); dmitriy.smolensky@usda.gov (D.S.); 5 Department of Pharmacy, School of Pharmacy, University of Salerno, 84084 Fisciano, Italy; gipepe@unisa.it (G.P.); pcampiglia@unisa.it (P.C.); 6 Bioteam Laboratory, Via Girolamo Santacroce, 80129 Napoli, Italy; felicita24@libero.it; 7 Sorghum Breeder, Fernando Camino N° 15 P4 F, 29016 Málaga, Spain; alberto.chessa@outlook.com; 8 DCSRSI SPR BIBLIOTECA, 80131 Napoli, Italy; mariarosaria.aletta@igb.cnr.it

**Keywords:** sorghum, pericarp, nutrition, grain, proximate composition, minerals, lipid composition

## Abstract

Grain sorghum (*Sorghum bicolor*) is a gluten-free cereal grown around the world and is a food staple in semi-arid and subtropical regions. Sorghum is a diverse crop with a range of pericarp colour including white, various shades of red, and black, all of which show health-promoting properties as they are rich sources of antioxidants such as polyphenols, carotenoids, as well as micro- and macro-nutrients. This work examined the grain composition of three sorghum varieties possessing a range of pericarp colours (white, red, and black) grown in the Mediterranean region. To determine the nutritional quality independent of the contributions of phenolics, mineral and fatty acid content and composition were measured. Minor differences in both protein and carbohydrate were observed among varieties, and a higher fibre content was found in both the red and black varieties. A higher amount of total saturated fats was found in the white variety, while the black variety had a lower amount of total unsaturated and polyunsaturated fats than either the white or red varieties. Oleic, linoleic, and palmitic were the primary fatty acids in all three analysed sorghum varieties. Significant differences in mineral content were found among the samples with a greater amount of Mg, K, Al, Mn, Fe, Ni, Zn, Pb and U in both red and black than the white sorghum variety. The results show that sorghum whole grain flour made from grain with varying pericarp colours contains unique nutritional properties.

## 1. Introduction

Sorghum (*Sorghum bicolor* (L.) Moench) is a widely consumed cereal staple in regions of Africa and Asia [1,2,3,4,5,6,7,8] and is the fifth leading cereal crop in the world, after the crops wheat, maize, rice, and barley [9]. The United States is the number one producer and exporter of sorghum, generating roughly 20% of total production and near 80% of total sorghum exports from 2001–2003 [10]). Where sorghum has been traditionally a basic food staple, it has also been used in several food products and in some cases health food items [8,11,12]. Sorghum does not contain peptide sequences that are toxic to persons with celiac disease, as are found in wheat, barley, and rye, and is therefore a safe food for celiac patients [13,14,15]. With increasing interest related to the unique properties of sorghum, its value as a food in helping to improve human health and to prevent disease has generated increasing research [1,2,4,11,15,16,17]. Specifically, increased research attention has focussed on the diverse content of phenolic compounds present in sorghum, which is a unique attribute among cereal grains [2]. These phenolic compounds have been shown to have various properties, e.g., inhibiting cancer cell growth [17], and while more research is needed on the health benefits of sorghum, consumption of whole grain sorghum may have the potential to help reduce health problems such as heart disease, diabetes, and obesity [16].

A current trend worldwide is a considerable preference for foods that have additional health benefits beyond basic nutrition. Research has continued to demonstrate that sorghum whole grains have numerous human health benefits, especially as related to antioxidant activity of phenolic compounds present in the outer layers of the grain [18,19]. The free radical scavenging activity of sorghum phenolic compounds has been related to beneficial health attributes, including anti-microbial properties [20], reduced oxidative stress [18], anti-inflammatory properties [21] and anti-cancer activity [17,22,23,24,25], thereby adding value to sorghum grains and its increasing human consumption [26]. The beneficial activities of sorghum for human health have been attributed mainly to the phenolic compounds found in sorghum grain and which are well known to vary with pericarp colour. While much of the research related to sorghum phenolic compounds and potential health benefits has been conducted using whole sorghum bran, or crude extracts from sorghum grain/bran, e.g., [6,17,18,20,21], numerous types of polyphenols have been identified in sorghum with examples including, flavonoids, hydroxybenzoic acids and hydroxycinnamic acids with specific levels varying according to both genetics and environment [24,27]. Several varieties of sorghum exist with a wide range of pericarp colour, and which can be classified based on the pigmentation of the pericarp [28]. In particular, research has shown that total phenolic content and antioxidant activity in sorghum are correlated with the pericarp thickness and colour, and sorghum with darker and thicker pericarp had greater levels of phenolic compounds and increased antioxidant activity [28,29]. Highly pigmented sorghum may therefore be desirable for use in human foods with improved human health attributes.

Substantial research has been conducted with the aim of developing the cultivation of sorghum lines in the Mediterranean area for use in production of human food products [8,30,31,32]. With that overall goal, the focus of this research was to compare the nutritional composition of sorghum varieties that differed in pericarp colour to (1) determine how nutritional properties other than phenolic content varied and (2) identify varieties with improved nutritional characteristics in addition to phenolic content and thus provide greater potential health value for consumers. Additionally, this research adds to the body of knowledge on sorghum grain nutrient composition, especially for sorghum grown outside the major sorghum producing regions.

## 2. Materials and Methods

### 2.1. Sorghum Cultivars

The sorghum varieties along with seed sources used in this research are shown in Table 1. In 2019, sorghum production was conducted in San Bartolomeo in Galdo (BN) in the Fortore area, which is in the Campania Region of southern Italy (41°25′ N, 15°01′ E and 597 m a.s.l.). The soil in this region is predominantly clay loam, deep and with a good water holding capacity. The milling was carried out starting from 1 month from the harvest of the sorghum grain, which was stored in a dry environment at 16 °C.

### 2.2. Flour Sample Preparation

Flour samples were produced from approximately 1 kg of grain samples that were milled using a two-roll mill (Chopin Moulin CD1; Chopin S.A., Villeneuve la Garenne, France) and subsequently were sieved using a planetary sieve (Buhler AG, Uzwil, Switzerland) with a screen size of 120 μm^2^.

### 2.3. Moisture

Moisture was determined according to the method described by Pontieri et al. [31]. Briefly, a ceramic capsule was accurately weighed after a complete desiccation at 100 °C in vacuum-packed (25 mm Hg) conditions using an oven (ISCO mod. NSV9035) and chilled at room temperature in a silica gel dryer. Then, an accurately weighed aliquot of flour samples (about 2 g) was placed in the desiccated ceramic capsule. The humidity was removed from the sample, by keeping it in the same temperature and pressure conditions for about five hours, until a constant weight was achieved. The moisture content was estimated by the weight loss.

### 2.4. Ash

To measure total ash, sorghum grain samples (ca. 3 g each) were weighed into broad, shallow ashing dishes and incinerated at ~550 °C, after which the dishes were placed in a desiccator to cool and then weighed after coming to room temperature [33].

### 2.5. Protein Content

Nitrogen content was measured using the Kjeldahl method [34] with total protein content determined with a conversion factor of 6.25. Sorghum grain samples (2 g each) were analysed with a Mineral Six Digester and an Auto Disteam semi-automatic distilling unit (International PBI, Milan, Italy).

### 2.6. Total Lipid Content

Total lipid content was measured as described by Pontieri et al. [30]. Briefly, approximately 3 g of grain was ground with liquid nitrogen using a mortar and pestle and lyophilized with the FTS-System Flex-DryTM instrument. The ground whole meal was then extracted using a Soxhlet apparatus with chloroform (CHCl_3_) for 4 h. Extracts were then dried with a rotary evaporator to obtain the crude extracts, which were subsequently weighed to determine the amount of extracted fat.

### 2.7. Gas Chromatography of Fatty Acids

Esterification of fatty acids from the crude extracts and subsequent gas chromatographic analysis of the fatty acid methyl esters was carried out as described previously [30,31]. Briefly, solid sorghum fat was melted in an oven at 50 °C to determinate its composition. A drop of fat was transferred into a 1.5 mL-vial. One ml of hexane and 100μL of 2N KOH methanolic solution were added. The vial was vortexed for 5 min, and then left under static conditions for 5 min, to enable a complete stratification of the hexanic portion, which contained the methyl ester of the fatty acids. Chromatographic separation was achieved using a GC-2010 (Shimadzu, Kyoto, Japan) equipped with a DB-Wax (Phenomenex, Torrance, CA, USA), 30 m length, 0.25 mm internal diameter, 0.25 µm film thickness column. The GC conditions were as follows: carrier gas, He; pressure, 75 kPa; injector temperature, 220 °C; FID temperature, 250 °C; and oven program, 170 °C for 8 min, 2°C/min to 185 °C for 10 min, 1 °C/min to 190 °C for 12 min, 10 °C/min to 240 °C for 5 min.

### 2.8. Carbohydrates

Carbohydrate content was determined by subtraction as the amount of material left after accounting for moisture, ash, protein, and fat content [35].

### 2.9. Fibre

Fibre was determined according to the AOAC [36] method. Briefly, fibre was determined as the loss, after incineration, of the sample digested in an acidic environment by H_2_SO_4_ (0.255 N), followed by an alkaline digestion with NaOH (0.223 N). Digestion was obtained with an automatic digestor (Velp Scientific mod. FIWE3, Usmate Velate, Monza e Brianza, Italy).

### 2.10. Total Minerals Determination

The determination of the mineral elements of interest was performed according to Tenore et al. [37] as described by Pontieri et al. [38].

Briefly, for each sample, the ash content was solubilized using ultrapure water (18 MΩ, produced using a Millipore Direct-Q UV3 water purifier) based HNO_3_ (Ultrapure, Sigma Aldrich, St. Louis, MO, USA) 5% solution. The solution was filtered using ash-free regenerated cellulose filters. All chemicals were of the highest commercially available purity grade. No glass (flask, pipettes, etc.) was used for any operation. Before use, all plastic containers were cleaned using 10% ultra-pure grade HNO_3_ for at least 24 h, and then rinsed copiously with ultra-pure water before use.

Element quantification was performed using quadrupole inductively coupled plasma mass spectrometry, ICP-QMS (820-MS, Bruker Daltonics, Billerica, MA). The operational parameters were: Plasma flow: 18 L/min, Auxiliary flow: 1.8 L/min, Sheath Gas: 0.14 L/min, Nebulizer flow: 0.98 L/min, RF power: 1.40 kW, Pump rate: 4 rpm, Stabilization delay: 20 s, First Extraction Lens: −40 volts, Second Extraction Lens: −166 volts, Third Extraction Lens: −234 volts, Corner Lens: −208 volts, Mirror Lens left: 29 volts, Mirror Lens right: 26 volts, Mirror Lens bottom: 30 volts; CRI parameters: Skimmer Gas: H_2_ at 50 mL/min, Sample Gas: He at 10 mL/min; dwell time, 10,000 µs; no. of scan replicate: 10, no. replicate for sample: 5. High purity He (99.9999% He, SALDOGAS Srl, Naples, Italy) and H_2_ (99.9999% H_2_, produced by the DBS H_2_ generator PGH2-300) were used, in order to minimize the potential problems caused by unidentified reactive contaminant species in the cell. Calibration solutions were prepared from multi-elemental standard stock solutions of 20.00 mg/L. Calibration curves were obtained using 9 calibration solutions. Reagent blanks containing ultra-pure water were additionally analysed to control the purity of the reagents and laboratory equipment. Standards and blanks were subjected to the same treatment as the samples. A mixed solution of internal standard (^6^Li, ^45^Sc, ^72^Ge, ^89^Y, ^103^Rh ^159^Tb, ^165^Ho, ^209^Bi) 10 µg/L was on-line aspired with a T union with the sample and standard solution.

### 2.11. ELISA Assay

The RIDASCREENR standard test kit [RIDASCREEN R Gliadin (Art. No R7001) R-Biopharm AG] sandwich ELISA based method was used to determine the presence of protein sequences reactive to gliadins in sorghum flour samples [39] following the manufacturer’s instructions. Commercial gliadin standard 16–18% N (Sigma Aldrich, Milan, Italy) was used as control.

### 2.12. Statistical Analysis

With the exception of total lipids analysis, which was performed in triplicate, all analyses were performed in quintuples (*n* = 5) (technical replicates), and the results are presented as the mean ± SD. Data distributions were evaluated by means of Shapiro–Wilk test. As all data was not normally distributed, differences in means were investigated using the non-parametric Mann–Whitney U test. Analysis of variance (ANOVA) was used to assess if the different values were statistically significant or not. The Tukey post-hoc test was used to identify which samples were different. False discovery rate (FDR) corrected *p*-value was used to manage the multiple comparisons.

## 3. Results

### 3.1. Nutrient Composition

The chemical composition of white, red, and black sorghum varieties developed in the USA but produced in Southern Italy is shown in Table 2. The table also reports the recommended daily dose (RDA) according to the European legislation [40]. Minor variations in both protein and carbohydrate were observed among the three coloured sorghum varieties analysed, while a higher fibre content was found in both the red and black varieties (*p* < 0.05).

### 3.2. Fatty Acid Composition of Total Lipids

The percentages of total fatty acids, also aggregated as saturated, mono-unsaturated and polyunsaturated fats, of white, red and black sorghum varieties are shown in Table 3. Greater levels of total saturated fats (* *p* < 0.05) was found in the white variety than in the red and black varieties, while the black variety had a lesser amount of total unsaturated and polyunsaturated fats (* *p* < 0.05) than both the white and red varieties.

Oleic, linoleic, and palmitic, were the primary fatty acids in all three of the sorghum varieties analysed, which is in agreement with previously reported results [31,41,42]. The percentage of palmitic acid in the black sorghum variety was slightly lower than both the white and red varieties, while the percentage of linoleic acid was slightly higher in the black variety than in the white and red varieties. Finally, the percentage of oleic acid was comparable between the three varieties of sorghum.

### 3.3. Mineral Content

Levels of minerals from the three sorghum varieties are reported in Table 4. Statistical analysis was not performed on the mineral content due to the number of minerals tested. However, the levels of macro-elements followed the sequence K > Mg > Ca > Na in all three varieties analysed. Micro-element content followed the sequence Fe > Zn > Al > Mn > Cr > Ni > Cu > Ba > Mo > Pb > Co > Sn > Ag > As > Se > V > Be > Tl in the white variety, while the content of micro-elements followed the sequence Fe > Zn > Al > Mn > Ni > Cu > Cr > Ba > Mo > Pb > Co > Sn > Ag > As > Se > V > Be > Tl in both the red and black varieties analysed. The white variety had a lower element content than that of both the red and black varieties, with K, Fe and Sb were the most abundant macro-element, micro-element, and trace element in all analysed varieties, except Hg which was the most abundant trace element in the white variety. The potassium and sodium content of the samples varied from 26.89 to 35.66 g kg^−1^ and 0.42 to 0.54 g kg^−1^, respectively, with the potassium content of the samples ranging from about 64-fold to 66-fold higher than that of sodium. Therefore, the K:Na ratio was higher than the recommended ratio 5.0 [43] for the human diet. The fact that the sorghum hybrids all contained a high K:Na ratio suggests that sorghum could be used to modulate sodium-related health problems. In fact, diets with a higher K:Na ratio are recommended for certain health conditions such as [44].

### 3.4. Immunochemical Evidence for the Absence of Gluten in Coloured Sorghum Varieties

Immunochemical measurement of gliadin concentration in the sorghum flour from all samples tested showed that gluten levels in all sorghum cultivars were less than 5 ppm (the detectable limit) (Table 5) and are at levels substantially below the 20 mg/kg (ppm) threshold recommended as safe for celiac patients [39].

## 4. Discussion

As it has been reported that pericarp colour of sorghum grain may vary due to both genotype environmental factors [42,45,46], in this work we compared both the chemical composition and the content of fatty acids and the mineral content of three coloured varieties of sorghum grown in the Mediterranean environment of Southern Italy. The search for varieties of sorghum developed in the USA that have high functional and nutraceutical properties when grown in the Mediterranean area will stimulate the use of sorghum for human use as a health food in European countries; it may encourage European farmers to produce sorghum, as it is a drought tolerant plant very well suited to environmental changes [8].

The composition profiles of white, red, and black food-grade sorghum varieties developed in the USA, and grown in Southern Italy, were overall similar with slight differences in both protein and carbohydrate percentages. The higher fibre content found in the red and black varieties suggests that this variety may have health benefits in addition to those conferred from just phenolic compounds. The black sorghum also had slightly higher total protein levels and less total fat, which could be minor benefits for use of black sorghum flour in human food products.

The quantities of the total saturated and mono-unsaturated fats of both the white and red varieties were similar and higher than those of the black variety, while the red and black varieties had similar quantities of total polyunsaturated fats but lower than that of the white variety. Thus, the black variety analysed in this research may have a slight nutritional advantage related to consumption of saturated fat relative the other two varieties. Oleic, linoleic, and palmitic were the primary fatty acids in all the sorghum varieties. Unsaturated fatty acids are important for human nutrition, as they are major components of biological membranes and play a role in modulating the fluidity of membranes. Additionally, unsaturated fatty acids do not have cholesterogenic properties (unlike saturated fatty acids), and reduce the risk of thrombosis, due to anti-aggregating activity of blood lipoprotein particles. Because of these features, unsaturated fatty acids are strongly recommended to lower the risk of atherosclerosis [4,11,16]. The sorghum samples analysed in this work all contained some levels of unsaturated fatty acids and could supplement other plant-based sources of unsaturated fats in human diets.

The content of each macro-element followed the sequence K > Mg > Ca > Na in all three coloured sorghum varieties analysed with the primary mineral being K, followed by Mg, which is consistent with the literature [38,47,48,49]. Furthermore, the concentrations of the above four macro-elements were higher in the red and black sorghum varieties than in the white sorghum variety, confirming previous works whose results indicate that the mineral content of sorghum was affected by both genetic and environmental factors [38]. With regards to macro-element content, this research reported a K:Na ratio greater than what is recommended in the human diet for all sorghum varieties analysed [43]. An improved K:Na ratio may improve bone health, lessen muscle loss, and moderate other chronic diseases such as hypertension and stroke [44]. In addition to the above, the magnesium content in the sorghum varieties was greater than typically found in corn (on average, 0.47 g kg^−1^) and wheat flour (on average, 0.25 g kg^−1^) [50]. Because each of the three types of coloured sorghum varieties analysed have high magnesium content, these sorghum varieties may be good sources of magnesium. Magnesium is an important macro-element because it is required for the function of many enzyme systems and therefore human metabolism [50].

The content of micro-elements followed the sequence Fe > Zn > Al > Mn > Cr > Ni > Cu > Ba > Mo > Pb > Co > Sn > Ag > As > Se > V >Be > Tl in the white variety analysed, while the content of micro-elements followed the sequence Fe > Zn > Al > Mn > Ni > Cu > Cr > Ba > Mo > Pb > Co > Sn > Ag > As > Se > V > Be > Tl in both red and black varieties analysed. The differences in the concentrations of some micro-elements between the white sorghum variety and both red and black sorghum varieties reported above could be affected by the sorghum variety, soil conditions and the state of plant maturity at harvest [38]. The most abundant micro-element was Fe in all three sorghum varieties analysed, confirming the data reported in the literature [38,46,49]. The latter is an essential micro-element in human nutrition, and Fe-deficiency is a major public health threat worldwide [6]. The expanding production of sorghum for human use in the US [11] and in Mediterranean countries [8], suggests the use of this cereal for healthy nutrition. Thus, identifying sorghum varieties with the highest levels of Fe is beneficial when identifying sorghum varieties for production in Europe.

The concentrations of trace element content followed the sequence Hg > Sb > Cd > U in the white variety, while it followed the sequence Sb > Hg > Cd > U in both red and black varieties. Importantly, with regards to the trace elements Sb, Hg, Cd, U, their concentration in the three sorghum hybrids analysed in this study did not exceed the maximum permitted by Regulation (CE) n. 41/2009.

Regarding the micro-elements content, the results reported in the present study show high content of both Fe and Zn in all sorghums. The latter two elements are essential micro-elements in human nutrition, and Fe and Zn deficiencies are worldwide public health issues [6].

Furthermore, in this work, the sorghum varieties developed in the USA and grown in the Mediterranean environment were also analysed immunochemically to measure the concentration of gliadin to verify previous reports on safety of sorghum for people with celiac disease. As shown in Table 5, the results indicated that the gluten levels in all sorghum cultivars were less than 5 mg/kg (below detectable limits) which is below the 20 mg/kg level proposed as a safe level for celiac patients [39] and agrees with previous results [13,14,15].

## 5. Conclusions

Consumers worldwide have increasingly expressed interest in both functional and nutraceutical foods due to the additional health benefits provided through their consumption. Substantial research has been focused on identifying the mechanisms associated with the disease prevention or therapeutic potential of such foods. One example of a functional and nutraceutical food that has received increased research attention is sorghum grain. It is well known that sorghum is a genetically diverse crop—that diversity extends to the presence of phenolic content and composition, and results in phenotypic expression in sorghum grain with a range of pericarp colours. Sorghum has been studied for several potential human health benefits, including the role of sorghum phenolic compounds present in types of sorghum that vary in pericarp colour. The present study supports the continued strategy of evaluating sorghum with a range of pericarp colour not only for the properties of their phenolic compounds, but also for additional nutritional properties such as protein and carbohydrate contents, levels of unsaturated fatty acids and minerals. Sorghum varieties developed for production in the USA and grown in the Mediterranean region demonstrate the feasibility of producing a range of different sorghums that vary in polyphenolic content and the high antioxidant capacity of the compound eriodictyol-O-hexoside isolated from the red sorghum variety, a flavonoid very important for human health due to its ability to fight free radicals with high efficiency [51]. The current research provides valuable information on nutrient composition of sorghum and supports the growing research on the unique health benefits of sorghum whole grain consumption. This research also shows that sorghum varying in pericarp colour and in associated phenolic compounds [50] can also vary in overall nutrient composition.

Cereals have long been consumed by humans and are staple foods providing a primary source of carbohydrates, proteins, B vitamins and minerals for a substantial portions of the world’s population; this is especially so where sorghum is consumed as the primary food source. Sorghum also contains a variety of phytochemicals which may, in addition to basic nutrition, provide some of the health benefits seen in populations consuming primarily plant food-based diets [47]. The fact that the nutritional composition was similar between the same varieties of sorghum grown in the USA and in the Mediterranean area is confirmation that it is possible to utilize sorghum for human use in Europe.

## Figures and Tables

**Table 1:** List of sorghum varieties.

Variety Colour	Variety Name	Source	Supplied by
White sorghum	DSM 3-410	Richardson Seeds Ltd.	M. Malin
Red sorghum	DSM IF-41912	Richardson Seeds Ltd.	M. Malin
Black sorghum	DSM 212-2311	Richardson Seeds Ltd.	M. Malin

**Table 2:** Nutritional values of white, red, and black sorghum varieties. Abbreviation: Recommended Daily dose (RDA).

Parameter	White	Red	Black	RDA
Moisture (%)	11.86 ± 0.06	11.92 ± 0.04 *	11.44 ± 0.09§	
Ash (%)	1.22 ± 0.04	1.44 ± 0.05 *	1.88 ± 0.06 *§	
Total proteins (%)	6.14 ± 0.10	6.85 ± 0.07 *	7.28 * ± 0.09 §	50 g/day
Fats (%)	2.23 ± 0.05	2.00 ± 0.04 *	1.55 * ± 0.03 §	70 g/day
Total carbohydrates (%)	73.17 ± 0.27	71.32 ± 0.30 *	70.07 * ± 0.39 §	260 g/day
Sugars (%)	0.67 ± 0.05	0.63 ± 0.06	0.77 ± 0.06	90 g/day
Fibres (%)	5.37 ± 0.14	6.46 ± 0.29 *	7.78 ± 0.24 *§	

FDR corrected *p*-value < 0.05 comparing to white sorghum was indicated as *, while comparing to red sorghum as §.

**Table 3:** Fatty acid content (g per 100 g raw fat) of white, red, and black sorghum varieties.

Fatty Acid	White	Red	Black
Myristic acid	0.013 ± 0.000	0.012 ± 0.000	0.036 ± 0.000 *§
Palmitic acid	18.633 ± 0.001	17.322 ± 0.01 *	12.769 ± 0.001 *§
Palmitoleic acid	0.824 ± 0.002	0.792 ± 0.002	0.690 ± 0.001 *§
Margaric acid	0.064 ± 0.534	0.065 ± 0.537	0.099 ± 0.385 *§
Margaroleic acid	0.063 ± 0.447	0.061 ± 0.421	0.067 ± 0.0438 *§
Stearic acid	2.226 ± 0.613	2.236 ± 0.598	1.234 ± 0.599 *§
Oleic acid	37.62 ± 0.031	42.655 ± 0.029 *	40.178 ± 0.018 *§
Linoleic acid	35.707 ± 0.062	33.985 ± 0.058	42.084 ± 0.052 *§
Linolenic acid	2.051 ± 0.087	1.958 ± 0.081	2.084 ± 0.091 *§
Arachidic acid	0.435 ± 0.026	0.374 ± 0.025 *	0.214 ± 0.028 *§
Eicosenoic acid	0.302 ± 0.023	0.274 ± 0.021 *	0.220 ± 0.022 *§
Behenic acid	0.072 ± 0.021	0.061 ± 0.019 *	0.027 ± 0.023 *§
Lignoceric acid	0.203 ± 0.015	0.188 ± 0.013 *	0.232 ± 0.018 *§
Erucic acid	0.019 ± 0.015	0.018 ± 0.013	0.065 ± 0.014 *§
Saturated fats	0.52 ± 0.05	0.41 ± 0.04 *	0.22 ± 0.03 *§
Monounsaturated fats	0.85 ± 0.02	0.89 ± 0.01	0.64 ± 0.02 *§
Polyunsaturated fats	0.86 ± 0.07	0.71 ± 0.02 *	0.69 ± 0.02 *

FDR corrected *p*-value < 0.05 comparing to white sorghum was indicated as *, while comparing to red sorghum as §.

**Table 4:** Elements content in sorghum varieties. Abbreviation: Recommended Daily dose (RDA).

Metal	Unit	White	Red	Black	RDA
K	g/Kg	26.89 ± 0.62	32.02 ± 0.68 *	35.66 ± 0.61 *§	2.0 g/day
Mg	g/Kg	8.69 ± 0.35	12.86 ± 0.22 *	16.93 ± 0.41 *§	0.375 g/day
Ca	g/Kg	1.25 ± 0.03	2.16 ± 0.03 *	2.88 ± 0.08 *§	0.8 g/day
Na	g/Kg	0.42 ± 0.04	0.43 ± 0.01	0.54 ± 0.01 *§	
Fe	mg/Kg	346.91 ± 1.35	578.75 ± 1.42 *	655.15 ± 1.37 *§	14 mg/day
Zn	mg/Kg	180.21 ± 0.86	250.47 ± 5.34 *	284.35 ± 7.59 *§	10 mg/day
Al	mg/Kg	54.59 ± 0.54	179.01 ± 5.43 *	200.01 ± 4.68 *§	
Mn	mg/Kg	49.81 ± 1.22	79.44 ± 2.58 *	98.68 ± 2.53 *§	2 mg/day
Cr	mg/Kg	31.17 ± 1.13	43.54 ± 1.55 *	53.74 ± 0.61 *§	0.04 mg/day
Ni	mg/Kg	29.32 ± 0.51	40.13 ± 0.78 *	49.91 ± 1.46 *§	
Cu	mg/Kg	24.11 ± 0.49	31.83 ± 0.88 *	35.56 ± 0.83 *§	1 mg/day
Ba	mg/Kg	4.63 ± 0.14	6.46 ± 0.25 *	8.00 ± 0.08 *§	
Mo	mg/Kg	1.15 ± 0.03	1.62 ± 0.02 *	2.05 ± 0.05 *§	0.05 mg/day
Pb	mg/Kg	0.57 ± 0.06	1.39 ± 0.04 *	1.61 ± 0.06 *§	
Co	mg/Kg	0.50 ± 0.02	0.96 ± 0.01 *	1.07 ± 0.01 *§	
Sn	mg/Kg	0.17 ± 0.01	0.31 ± 0.01 *	0.39 ± 0.01 *§	
Ag	mg/Kg	0.17 ± 0.01	0.30 ± 0.01 *	0.34 ± 0.01 *§	
As	mg/Kg	0.63 ± 0.01	0.23 ± 0.01 *	0.28 ± 0.01 *§	
Se	mg/Kg	0.08 ± 0.01	0.05 ± 0.01 *	0.07 ± 0.01 *§	0.055 mg/day
V	mg/Kg	<0.01	<0.01	<0.01	
Be	mg/Kg	<0.01	<0.01	<0.01	
Tl	mg/Kg	<0.01	<0.01	<0.01	
Sb	μg/Kg	38.62 ± 0.90	64.69 ± 1.10 *	79.80 ± 2.01 *§	
Hg	μg/Kg	61.56 ± 3.08	50.81 ± 2.06 *	62.70 ± 2.20 §	
Cd	μg/Kg	11.86 ± 0.20	32.90 ± 0.68 *	36.93 ± 0.86 *§	
U	μg/Kg	3.14 ± 0.05	5.54 ± 0.13 *	6.21 ± 0.12 *§	

FDR corrected *p*-value < 0.05 comparing to white sorghum was indicated as *, while comparing to red sorghum as §.

**Table 5:** Measurement of gliadin (as ppm) in flours by using sandwich R5 enzyme-linked immunosorbent assay (ELISA).

Variety	Type	Content (ppm) ^2^
White	Sorghum	<5
Red	Sorghum	<5
Black	Sorghum	<5
Gliadin standard ^1^	Wheat	56

^1^ Gliadin standard from wheat (Sigma). ^2^ Mean values from 3 measurements.

## Data Availability

All the data are reported in the paper.
